# Molecular characterization of tick-borne bacterial and protozoan pathogens in parasitic ticks from Xinjiang, China

**DOI:** 10.1186/s13071-025-06857-1

**Published:** 2025-06-04

**Authors:** Bingjie Wang, Zhiqiang Liu, Shiying Zhu, Jinchao Zhang, Wenwen Qi, Jianyu Wang, Dongfang Li, Lan He, Junlong Zhao

**Affiliations:** 1 National Key Laboratory of Agricultural Microbiology, College of Veterinary Medicine, Huazhong Agricultural University, Wuhan, 430070 Hubei China; 2 Key Laboratory of Preventive Veterinary Medicine in Hubei Province, The Cooperative Innovation Center for Sustainable Pig Production, Wuhan, 430070 Hubei China; 3 Veterinary Research Institute, Xinjiang Academy of Animal Sciences (Animal Clinical Medical Research Center, Xinjiang Academy of Animal Sciences), Urumqi, 830013 Xinjiang China; 4 Animal Husbandry and Veterinary Station of the Urumqi District, Urumqi, 830000 Xinjiang China; 5 Key Laboratory of Development of Veterinary Diagnostic Products, Ministry of Agriculture of The People’s Republic of China, Wuhan, 430070 China

**Keywords:** Tick species, Tick-borne pathogens, *Anaplasma*, *Ehrlichia*, *Rickettsia*, *Borrelia burgdorferi*, Piroplasm, Xinjiang

## Abstract

**Background:**

Ticks are a type of hematophagous parasite, serving as critical vectors of pathogens that cause numerous human and animal diseases. Climate change has driven the geographical expansion of tick populations and increased the global transmission risk of tick-borne diseases. However, there has been a lack of comprehensive data on tick species distribution and their associated pathogen profiles in Xinjiang, China.

**Methods:**

Ticks were collected from 19 sampling sites across nine regions in Xinjiang. The species were identified using both morphological and molecular biological methods. The presence of tick-borne bacterial and protozoan pathogens was detected through polymerase chain reaction (PCR). Finally, sequencing and phylogenetic analyses were performed to further characterize the identified ticks and pathogens.

**Results:**

A total of 1093 ticks were collected and identified, representing four genera and nine species, with *Hyalomma asiaticum* being the dominant species. Haplotype diversity and genetic differentiation analysis based on the *16S rRNA* gene of the dominant species demonstrated that the *Hy. asiaticum* population in Xinjiang exhibits high haplotype diversity (Hd = 0.734), low nucleotide diversity (*π* = 0.00403), and significant genetic differentiation (Fst = 0.19716). Pathogen detection using PCR revealed an infection rate of 9.3% for *Anaplasma*, 18.1% for *Rickettsia*, and 9.0% for piroplasms. Phylogenetic analysis based on *16S rRNA* sequences indicated that the *Anaplasma* genus identified in ticks comprised *Anaplasma ovis*, *Anaplasma* sp., and *Anaplasma phagocytophilum*. Phylogenetic analysis based on the *opmA* gene showed that the *Rickettsia* genus identified in ticks included *Rickettsia aeschlimannii*, *Rickettsia conorii*, *Rickettsia slovaca*, *Rickettsia conorii* subsp. *raoultii*, *Rickettsia* sp., *Candidatus Rickettsia barbariae*, and *Candidatus Rickettsia jingxinensis*. Similarly, phylogenetic analysis based on the *18S rRNA* gene demonstrated that the piroplasms identified in ticks included *Theileria annulata*, *Theileria ovis*, *Babesia bigemina*, *Babesia occultans*, and *Babesia* sp. All gene sequences of the detected pathogens showed 99.8–100% identity with corresponding sequences deposited in GenBank.

**Conclusions:**

This study demonstrates that Xinjiang harbors a rich diversity of tick species with a wide geographical distribution. Furthermore, the tick-borne pathogens in this region are complex and diverse. These results underscore the necessity of sustained and enhanced surveillance efforts targeting ticks and tick-borne diseases in this region

**Graphical abstract:**

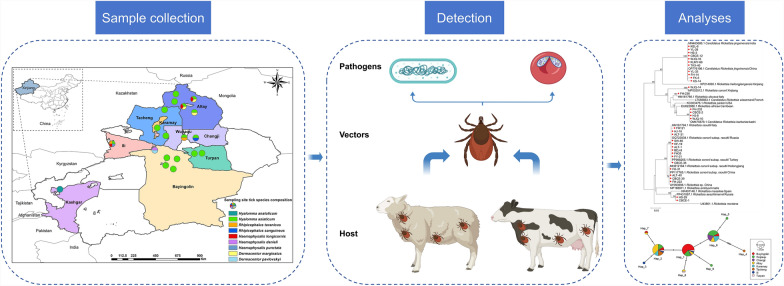

**Supplementary Information:**

The online version contains supplementary material available at 10.1186/s13071-025-06857-1.

## Background

Ticks are a type of obligate hematophagous arthropod with a broad range of hosts and can parasitize birds, amphibians, and nearly all terrestrial vertebrates, including humans [1, 2]. Epidemiologically, they are recognized as the second most significant vector of human pathogens only next to mosquitoes and also primary vectors for zoonotic disease transmission in animals [3]. During blood-feeding, ticks inflict mechanical damage to the hosts and simultaneously transmit various pathogens, causing severe zoonotic infections and therefore posing substantial risks to public health and livestock industry [4]. Since 1982, 33 tick-associated pathogens, including viruses, bacteria, and protozoa, have been identified in mainland China [5].

*Rickettsia*, *Anaplasma*, and *Ehrlichia* are gram-negative intracellular symbionts and significant tick-borne pathogens. *Rickettsia* spp. can be classified into four major groups: spotted fever group (SFG), typhus group (TG), ancestral group (AG), and transitional group (TRG). Among these groups, the TG and SFG harbor numerous clinically significant human pathogens. For instance, *Rickettsia prowazekii* and *Rickettsia typhi* in the TG group are causative agents of epidemic typhus and endemic typhus, respectively. The SFG group, as the largest taxon in the *Rickettsia* genus, mainly comprises pathogens responsible for Rocky Mountain spotted fever (*Rickettsia rickettsii*), Japanese spotted fever (*Rickettsia japonica*), Mediterranean spotted fever (*Rickettsia conorii*), and African tick bite fever (*Rickettsia africae*) [6, 7]. *Anaplasma* and *Ehrlichia*, which belong to the Rickettsiales order and Anaplasmataceae family, carry various animal pathogens, such as *Anaplasma marginale*, *Anaplasma centrale*, *Anaplasma bovis*, *Ehrlichia minasensis*, and *Ehrlichia canis* [8, 9]. Notably, several of these pathogens exhibit zoonotic potential; for example, *Anaplasma phagocytophilum* causes human granulocytic anaplasmosis (HGA), while *Ehrlichia chaffeensis* is associated with human monocytic ehrlichiosis (HME) [10]. A newly identified species, *Anaplasma capra*, represents newly emerging risks, as it can induce febrile syndromes with headache and fatigue in humans [11].

Piroplasmosis is a tick-borne protozoan disease caused by *Babesia* spp. and *Theileria* spp., posing significant threats to both domestic livestock (such as cattle, sheep, horses) and wildlife, with occasional zoonotic spillover to humans [12]. The clinical symptoms of piroplasmosis include anemia, hyperthermia, jaundice, and hemoglobinuria. In severe cases, it can lead to the death of the host, causing significant economic losses to the livestock industry [13, 14]. In China, piroplasmosis is endemic across diverse regions. Bovine theileriosis is prevalent in Xinjiang, Ningxia, and Inner Mongolia. Concurrently, bovine babesiosis has been reported in 21 provinces of China, particularly in Xinjiang, Gansu, Inner Mongolia, Tibet, Henan, Shaanxi, and Yunnan [15].

Xinjiang is situated on the northwestern border of China and geographically central to the Eurasian continent, representing one of the China’s five major pastoral regions [16]. Owing to its vast territories, diverse geographical landscapes, and rich biodiversity in forest–grassland ecosystems, Xinjiang provides ideal habitats for ticks, making it a hotspot for tick species diversity (48 species across eight genera and two families, accounting for approximately one third of all tick species documented in China) and a high-risk zone for tick-borne diseases [17]. To date, various tick-borne diseases have been reported in Xinjiang, including tick-borne encephalitis (TBE), Crimean–Congo hemorrhagic fever (CCHF), Lyme borreliosis, spotted fever group rickettsioses, and piroplasmosis [18]. The spread and prevalence of both traditional and newly emerging tick-borne diseases are further exacerbated by extensive border with eight countries and frequent international livestock trade in this region [19]. Since there has been limited research on tick-borne diseases in neighboring countries, it is crucial to conduct epidemiological surveys and continuous monitoring of tick-borne pathogens in Xinjiang for better disease prevention and control in this region. In this study, we collected ectoparasitic ticks from livestock farms across different ecoregions in Xinjiang to investigate the prevalence and genetic diversity of tick-borne bacterial and protozoan pathogens. The findings are expected to facilitate informed and targeted control of tick-borne diseases and alleviate zoonotic threats in this region.

## Methods

### Collection and species identification of tick samples

**Fig. 1:**
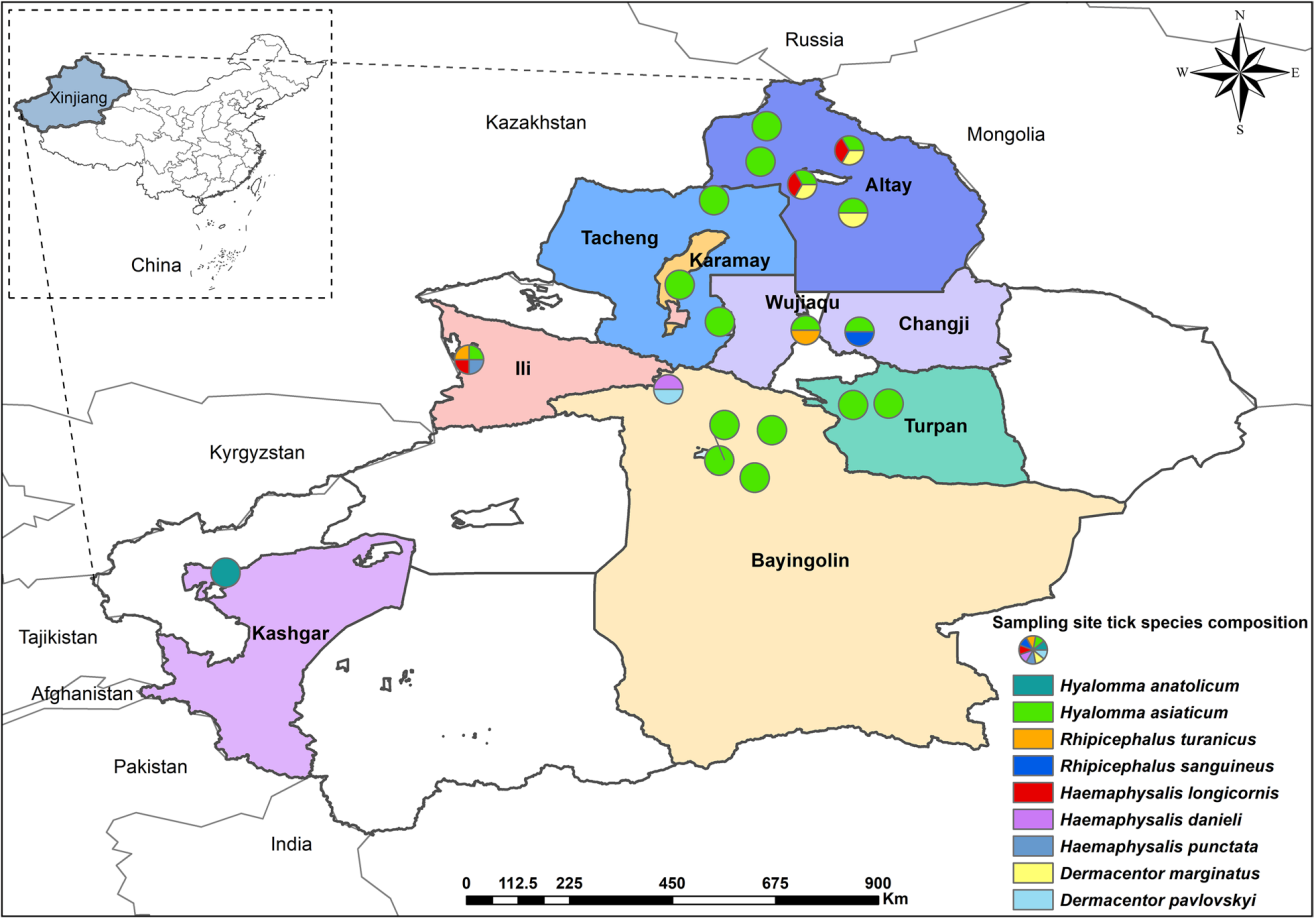
Ticks collected across various regions in Xinjiang, China. In the figure, circular markers indicate sampling sites, with each color corresponding to a distinct tick species

From May to June in 2023 and 2024, a total of 1093 adult ticks, including both blood-fed and unfed ticks, were randomly collected from the body surfaces of free-ranging mammalian hosts at 19 sampling sites across nine regions in Xinjiang, including Altay, Tacheng, Ili, Karamay, Changji, Wujiaqu, Turpan, Bayingolin, and Kashgar (Fig. 1). The ticks were collected from various body parts of the hosts, including the ears, periocular, neck, perineum, perianal area, and the base of the tail [20].

Tick species were initially identified morphologically under a stereo microscope (Nikon, Shanghai, China) with a taxonomic key on the basis of features such as basis capituli, scutum, anal groove, and peritreme [21, 22] and then confirmed by polymerase chain reaction (PCR) amplification and sequencing of the *16S rRNA* gene (Additional File 1: Supplementary Table S1) [23].

### DNA extraction

All the ticks were sterilized by immersion in 70% ethanol, washed three times with sterile phosphate-buffered saline (PBS; pH 7.2–7.4), dried on filter papers, and collected in individual sterile microtubes. Ticks were individually homogenized using Tissuelyser-24 (Jingxin, Shanghai, China) with 3.0 mm stainless steel beads in 200 μl of PBS. Genomic DNA was extracted from the homogenates using TIANamp Tissue and Blood Kit (Tiangen, Beijing, China) and stored at –20 ℃ according to the manufacturer’s instructions.

### Molecular detection of tick-borne pathogens

Bacterial pathogens (*Anaplasma*, *Ehrlichia*, *Rickettsia*, and *Borrelia burgdorferi*) and protozoan pathogens (*Theileria* and *Babesia*) were screened using conventional PCR, nested PCR, or semi-nested PCR with the corresponding primers described in previous studies (Additional file 1: Supplementary Table S1) [24–27]. Nuclease-free water was used as the negative control set in each PCR assay. PCR reactions were performed in a T100™ Thermal cycler (Applied Bio-Rad, CA, USA), using the following conditions: initial denaturation at 95 ℃ for 3 min; followed by 35 cycles of denaturation at 95 ℃ for 15 s, annealing for 15 s and extension at 72 ℃ for 15 s/kb; and a final extension at 72 ℃ for 5 min. Annealing temperatures are listed in Additional file 1: Supplementary Table S1. PCR products (5 μl) were analyzed by 1.5% agarose gel electrophoresis. The target amplicons were sent to Sangon Biotech (Shanghai, China) for bi-directional Sanger sequencing.

### Sequence analysis

The obtained sequences were checked and assembled using Chromas 2.6.6 and SeqMan 7.1 (DNASTAR, Madison, Wisconsin, USA), which were then aligned with sequences deposited in GenBank database by BLASTn search to determine the nucleotide identities and similarities. Multiple sequence alignment was performed using Clustal X2 [28] and BioEdit 7.0.9.1 [29]. The phylogenetic trees were constructed using the neighbor-joining (NJ) algorithm on the basis of the best-fit substitution model determined by MEGA 7.0 software with 1000 bootstrap replicates to assess tree stability. The target gene sequences of ticks as well as *Anaplasma* spp., *Rickettsia* spp., *Theileria* spp., and *Babesia* spp. detected in ticks (Additional file 1: Supplementary Table S2) and different reference sequences in GenBank (Additional file 1: Supplementary Table S3) were used for genetic evolution analysis. *Sarcoptes scabiei* (accession no. AF387675.1) was used as an outgroup of the tick phylogenetic tree. *R. conorii* (accession no. L36107.1) was included in the tree of *Anaplasma* as the outgroup. To analyze the genetic diversity of the dominant tick species, DnaSP version 6.10.04 software was used to quantify the number of haplotypes (Hn) and calculate the haplotype diversity (Hd) and nucleotide diversity (*π*) [30]; PopART version 1.7 was used to construct a haplotype network [31]; furthermore, Arlequin version 3.5.2.2 was used to calculate the genetic differentiation index (F statistics, Fst); and a neutrality test was performed to obtain Fu’s Fs and Tajima’s D values [32].

### Statistical analysis

IBM SPSS Statistics 20.0 software was used for statistical analysis. The difference in infection rate by *Anaplasma*, *Rickettsia*, and piroplasms among different regions was compared by Pearson’s chi-squared (*χ*^2^) test. Significant differences were determined at *P* < 0.05.

## Results

### Identification of tick species

A total of 1093 ticks were collected and subjected to morphological and molecular identification, which were identified as four genera and nine species, namely *Hyalomma anatolicum*, *Hyalomma asiaticum*, *Dermacentor marginatus*, *Dermacentor pavlovskyi*, *Rhipicephalus turanicus*, *Rhipicephalus sanguineus*, *Haemaphysalis longicornis*, *Haemaphysalis punctata*, and *Haemaphysalis danieli*. *Hy. asiaticum* (72.0%, 787/1093) was the dominant tick species, while *Ha. danieli* (0.4%, 4/1093) was the rarest species (Table 1). Among the nine regions sampled, the largest number of ticks was collected in the Altay region (37.2%, 407/1093); the smallest number of ticks was collected in the Kashgar region (2.4%, 26/1093); while the richest tick species were collected in the Ili region (Table 1).

**Table 1:** Species identification and quantity statistics of ticks from nine regions in Xinjiang

Region	Sampling site	Geographical coordinates (°N, °E)	Geographical inhabitants		Host	Tick species	Number	Total
			Altitude (m)	Habitat types				
Kashgar	Kashgar	39.5, 76.1	1267	Desert, Gobi	Cattle	*Hy. anatolicum*	26	26
Bayingolin	Korla^a^	41.7, 85.9 41.7, 85.8	903–908	Plain	Sheep	*Hy. asiaticum*	63	151
	Yuli	41.4, 86.2	889	Desert, Gobi	Cattle		42	
	Hoxud	42.3, 86.9	1122	Montane grassland	Cattle, sheep		30	
	Hejing	43.1, 84.8	2511	Alpine frigid grassland	Sheep	*Ha. danieli*	4	
						*D. pavlovskyi*	12	
Turpan	Toksun^a^	42.8, 88.6 42.8, 88.5	134–138	Desert steppe	Cattle	*Hy. asiaticum*	115	115
Wujiaqu	Wujiaqu	44.2, 87.5	460	Plain grassland	Cattle, sheep	*Hy. asiaticum*	94	136
						*R. turanicus*	42	
Changji	Fukang	44.2, 88.6	580	Plain grassland	Sheep	*Hy. asiaticum*	44	50
						*R. sanguineus*	6	
Karamay	Karamay	45.1, 85.1	293	Desert steppe	Cattle	*Hy. asiaticum*	78	78
Tacheng	Shawan	44.4, 85.8	409	Desert steppe	Cattle	*Hy. asiaticum*	70	89
	Hefeng	46.8, 85.7	1295	Desert steppe	Sheep		19	
Ili	Qapqal	43.7, 80.9	890	Mountain grassland	Sheep	*Hy. asiaticum*	8	41
						*R. turanicus*	17	
						*Ha. longicornis*	10	
						*Ha. punctata*	6	
Altay	Altay	47.8, 88.4	897	Mountain grassland	Cattle, sheep	*Hy. asiaticum*	34	407
						*Ha. longicornis*	1	
						*D. marginatus*	52	
	Fuyun	46.6, 88.5	656	Meadow steppe		*Hy. asiaticum*	41	
						*D. marginatus*	1	
	Fuhai	47.1, 87.5	499	Composite area of river valley and oasis		*Hy. asiaticum*	126	
						*Ha. longicornis*	11	
						*D. marginatus*	118	
	Burqin	47.9, 86.6	480	Meadow steppe		*Hy. asiaticum*	17	
	Habahe	48.3, 86.8	512	Composite area of river valley and oasis			6	
Total	–	–	–		*–*	*–*	1093	

^a^Korla and Torkun each include two sampling sites

### Detection of bacterial pathogens in ticks

*Anaplasma* and *Rickettsia* were detected in the ticks, while *Ehrlichia* or *B. burgdorferi* was not detected. Semi-nested PCR targeting the *16S rRNA* gene revealed an overall *Anaplasma* infection rate of 9.3% (102/1093). *Anaplasma* infection was the most prevalent in the Kashgar region, while the least prevalent in the Turpan region (Table 2). There were statistically significant differences (*χ*^2^ = 97.217, *df* = 8, *P* < 0.01) in the prevalence of tick infection among different regions (Table 2). The amplified fragment size of the positive samples was 650 base pairs (bp).

**Table 2:** Prevalence of tick-borne bacterial and piroplasmosis pathogens in ticks from nine regions of Xinjiang

Region	Sampling site	Number of examined ticks	Number of infected ticks (%, 95% confidence interval (CI))		
			Piroplasms	*Anaplasma*	*Rickettsia*
Kashgar	Kashgar	26	20 (76.9%)	7 (26.9%)	4 (15.4%)
Bayingolin	Korla	63	0 (0.0%)	10 (15.9%)	20 (31.7%)
	Yuli	42	2 (4.8%)	12 (28.6%)	14 (33.3%)
	Hoxud	30	9 (30.0%)	13 (43.3%)	3 (10.0%)
	Hejing	16	2 (12.5%)	0 (0.0%)	2 (12.5%)
Turpan	Toksun	115	2 (1.7%)	2 (1.7%)	7 (6.1%)
Wujiaqu	Wujiaqu	136	1 (0.7%)	5 (3.7%)	35 (25.7%)
Changji	Fukang	50	7 (14.0%)	12 (24.0%)	4 (8.0%)
Karamay	Karamay	78	4 (5.1%)	15 (19.2%)	3 (3.8%)
Tacheng	Shawan	70	2 (2.9%)	6 (8.6%)	1 (1.4%)
	Hefeng	19	0 (0.0%)	0 (0.0%)	1 (5.3%)
Ili	Qapqal	41	4 (9.8%)	6 (14.6%)	7 (17.1%)
Altay	Altay	87	11 (12.6%)	2 (2.3%)	26 (29.9%)
	Fuyun	42	5 (11.9%)	4 (9.5%)	1 (2.4%)
	Fuhai	255	29 (11.4%)	8 (3.1%)	69 (27.1%)
	Burqin	17	0 (0.0%)	0 (0.0%)	1 (5.9%)
	Habahe	6	0 (0.0%)	0 (0.0%)	0 (0.0%)
Total	–	1093	98 (9.0%, 7.3–10.7%)	102 (9.3%, 7.6–11.1%)	198 (18.1%, 15.8–20.4%)
Chi-squared	–	–	175.870	97.217	60.988
*P*-value	–	–	< 0.01	< 0.01	< 0.01

To detect *Rickettsia*, all tick samples were screened using semi-nested PCR targeting the *ompA* gene, and the results showed an overall infection rate of 18.1% (198/1093). The positive samples displayed an expected amplicon size of 533 bp. Notably, the prevalence of *Rickettsia* infection was the highest in the Kashgar region, whereas it was the lowest in the Turpan region (Table 2). Statistically significant differences (*χ*^2^ = 60.988, *df* = 8, *P* < 0.01) in infection rates were observed among various regions (Table 2).

### Detection of piroplasms in ticks

Conventional PCR targeting the *18S rRNA* gene showed that the overall prevalence of piroplasms in ticks across all regions was 9.0% (98/1093). The carriage rate of piroplasms in ticks from various regions followed a descending order of Kashgar (76.9%); Changji (14.0%); Altay (11.1%); Ili (9.8%); Bayingolin (8.6%); Karamay (5.1%); Tacheng (2.2%); Turpan (1.7%); and Wujiaqu (0.7%; Table 2). Statistical analysis indicated significant differences in piroplasm infection rates among different regions (*χ*^2^ = 175.870, *df* = 8, *P* < 0.01), as presented in Table 2. Positive samples exhibited an expected amplicon size of 1600 bp during PCR amplification.

### Analysis of tick-borne pathogen infection status under the influence of different factors

To investigate the impact of blood-feeding status of ticks on the prevalence of tick-borne pathogens, the 1093 collected ticks were grouped into blood-fed ticks (*n* = 319) and unfed ticks (*n* = 774) according to their physiological feeding states. Pathogen detection revealed that the prevalences of *Anaplasma* spp., *Rickettsia* spp., and piroplasms were 10.3% (33/319); 21.3% (68/319); and 9.7% (31/319) in blood-fed ticks, while 8.9% (69/774); 16.8% (130/774); and 8.7% (67/774) in unfed ticks, respectively.

Chi-squared tests showed no statistically significant differences in the prevalence of *Anaplasma* (*χ*^2^ = 0.546, *df* = 1, *P* > 0.05), *Rickettsia* (*χ*^2^ = 3.112, *df* = 1, *P* > 0.05), or piroplasms (*χ*^2^ = 0.312, *df* = 1, *P* > 0.05) between blood-fed and unfed ticks (Table 3).

**Table 3:** Pathogen infection prevalences in ticks across different blood-feeding status

Hematophagous state	Number of examined ticks	Number of infected ticks (%)		
		Piroplasms	*Anaplasma*	*Rickettsia*
Blood-fed	319	31 (9.7%)	33 (10.3%)	68 (21.3%)
Unfed	774	67 (8.7%)	69 (8.9%)	130 (16.8%)
Total	1093	98 (9.0%)	102 (9.3%)	198 (18.1%)
Chi-squared	–	0.312	0.546	3.112
*P*-value	–	> 0.05	> 0.05	> 0.05

Given that different tick-borne pathogens typically rely on specific tick species as primary transmission vectors, we analyzed the correlation between tick species and pathogen prevalence. The results showed that *Hy. asiaticum* harbored the greatest variety of pathogens (11 species), whereas no pathogen was detected in *Ha. danieli*. Analysis of *Anaplasma* infection in different tick species revealed that *A. ovis* was detected in five tick species, with no significant differences in prevalence among different tick species (*χ*^2^ = 13.999, *df* = 8, *P* > 0.05). *Anaplasma* sp. was only found in *Hy. anatolicum* and *Hy. asiaticum* and exhibited significant differences in prevalence among different tick species (*χ*^2^ = 47.957, *df* = 8, *P* < 0.01). *A. phagocytophilum* was only detected in *Hy. asiaticum* (Table 4). Analysis of *Rickettsia* infection in different tick species showed that *Rickettsia raoultii*, *Candidatus Rickettsia barbariae*, and *Candidatus Rickettsia jingxinensis* were detected in multiple tick species; *R. conorii*, *Rickettsia slovaca*, and *Rickettsia* sp. were only found in single tick species. Except for *Rickettsia* sp., the infection rate of six *Rickettsia* species, including *Rickettsia aeschlimannii* and *R. conorii*, showed significant differences among different tick species (Table 5). Analysis of piroplasm infection in different tick species revealed that *Theileria ovis* was prevalent in four tick species; *Theileria annulata* was exclusively detected in *Hyalomma* spp.; and each of *Babesia bigemina*, *Babesia occultans*, and *Babesia* sp. was restricted to a single tick species. Significant differences were found in the infection rate of *T. annulata*, *T. ovis*, and *B. occultans*, while not in that of *B. bigemina* and *Babesia* sp. among different tick species (Table 6).

**Table 4:** *Anaplasma* prevalences across different tick species

Tick species	Number of examined ticks	Number of infected ticks (%)		
		*A. ovis*	*Anaplasma* sp.	*A. phagocytophilum*
*Hy. anatolicum*	26	0	7 (26.9)	0
*Hy. asiaticum*	787	40 (5.1)	37 (4.7)	6 (0.8)
*D. marginatus*	171	6 (3.5)	0	0
*D. pavlovskyi*	12	0	0	0
*R. turanicus*	59	3 (5.1)	0	0
*R. sanguineus*	6	2 (33.3)	0	0
*Ha. longicornis*	22	1 (4.5)	0	0
*Ha. punctata*	6	0	0	0
*Ha. danieli*	4	0	0	0
Total	1093	52 (4.8)	44 (4.0)	6 (0.5)
Chi-squared	–	13.999	47.957	2.346
*P* value	–	> 0.05	< 0.01	> 0.05

**Table 5:** *Rickettsia* prevalences across different tick species

Tick species	Number of examined ticks	Number of infected ticks (%)						
		*R. aeschlimannii*	*R. conorii*	*R. slovaca*	*R. raoultii*	*Rickettsia* sp.	*Candidatus R. barbariae*	*Candidatus* *R. jingxinensis*
*Hy. anatolicum*	26	0	0	0	0	0	0	4 (15.4)
*Hy. asiaticum*	787	1 (0.1)	0	0	28 (3.6)	5 (0.6)	0	82 (10.4)
*D. marginatus*	171	0	0	7 (4.1)	45 (26.3)	0	0	0
*D. pavlovskyi*	12	0	0	0	1 (8.3)	0	1 (8.3)	0
*R. turanicus*	59	0	11 (18.6)	0	0	0	6 (10.2)	0
*R. sanguineus*	6	0	0	0	0	0	0	1 (16.7)
*Ha. longicornis*	22	0	0	0	3 (13.6)	0	2 (9.1)	0
*Ha. punctata*	6	1 (16.7)	0	0	0	0	0	0
*Ha. danieli*	4	0	0	0	0	0	0	0
Total	1093	2 (0.2)	11 (1.0)	7 (0.6)	77 (7.0)	5 (0.5)	9 (0.8)	87 (8.0)
Chi-squared	–	89.942	194.740	37.986	120.732	1.953	98.111	32.772
*P* value	–	< 0.01	< 0.01	< 0.01	< 0.01	> 0.05	< 0.01	< 0.01

**Table 6:** Piroplasm prevalences across different tick species

Tick species	Number of examined ticks	Number of infected ticks (%)				
		*T. annulata*	*T. ovis*	*B. bigemina*	*B. occultans*	*Babesia* sp.
*Hy. anatolicum*	26	20 (76.9)	0	0	0	0
*Hy. asiaticum*	787	30 (3.8)	25 (3.2)	2 (0.3)	0	4 (0.5)
*D. marginatus*	171	0	13 (7.6)	0	0	0
*D. pavlovskyi*	12	0	2 (16.7)	0	0	0
*R. turanicus*	59	0	0	0	1 (1.7)	0
*R. sanguineus*	6	0	1 (16.7)	0	0	0
*Ha. longicornis*	22	0	0	0	0	0
*Ha. punctata*	6	0	0	0	0	0
*Ha. danieli*	4	0	0	0	0	0
Total	1093	50 (4.6)	41 (3.8)	2 (0.2)	1 (0.1)	4 (0.4)
Chi-squared	–	326.230	20.621	0.779	17.541	1.561
*P* value	–	< 0.01	< 0.01	> 0.05	0.01–0.05	> 0.05

### Sequencing and phylogenetic analysis of tick species

All tick species were subjected to PCR amplification using specific primers targeting the *16S rRNA* gene, yielding a 460 bp fragment consistent with the expected size of the *16S rRNA* gene. Subsequent sequencing and BLASTn analysis in the National Center for Biotechnology Information (NCBI) database confirmed that the results were aligned with those of morphological identification.

Ticks collected from the Kashgar region were exclusively identified as *Hy. anatolicum*, exhibiting 100% identity with the Turkish strain (KR870971.1). In the Bayingolin region, the ticks included *Hy. asiaticum*, *Ha. danieli*, and *D. pavlovskyi*, showing 99.3–99.8% and 100% identity with the *Hy. asiaticum* strain from Kazakhstan (OR486027.1) and Xinjiang (MK530106.1, OR452926.1), respectively; 99.3% identity with the Chinese strain of *Ha. danieli* (NC_062065.1); and 99.1% identity with the Hebei strain of *D. pavlovskyi* (OK493294.1). Ticks from the Turpan region were all identified as *Hy. asiaticum*, with 99.8% identity to the Xinjiang strain (MG021188.1). In Wujiaqu, the ticks comprised *Hy. asiaticum* and *R. turanicus*, which displayed 99.1–99.5% and 100% identity with the *Hy. asiaticum* strain from Kazakhstan (OR486027.1) and Xinjiang (MG021188.1), respectively, and 99.3–100% identity with *R. turanicus* strains from Xinjiang (MT254805.1, KY583073.1). Ticks from Changji included *Hy. asiaticum* and *R. sanguineus*, with 99.5% and 100% identity to strains from Kazakhstan (OR486027.1) and Xinjiang (KU183525.1), respectively. Ticks from Karamay were all *Hy. asiaticum*, showing 99.5% identity to the Kazakhstan strain (OR486027.1). In Tacheng, all ticks were *Hy. asiaticum*, with 99.8–100% identity to the Xinjiang strain (MG021188.1). Ticks from Ili included *Hy. asiaticum*, *R. turanicus*, *Ha. longicornis*, and *Ha. punctata*, exhibiting 100%, 99.5%, 100%, and 99.8% identity to the strains from Kazakhstan (OR486027.1), Xinjiang (KY583073.1), Nanjing, and Xinjiang (MF002566.1), respectively. Finally, ticks from Altay comprised *Hy. asiaticum*, *Ha. longicornis*, and *D. marginatus*, with 99.5–99.8%, 100%, and 100% identity to the strains from Kazakhstan (OR486027.1), Nanjing (PP486235.1), and Kazakhstan (OR486023.1), respectively.

**Fig. 2:**
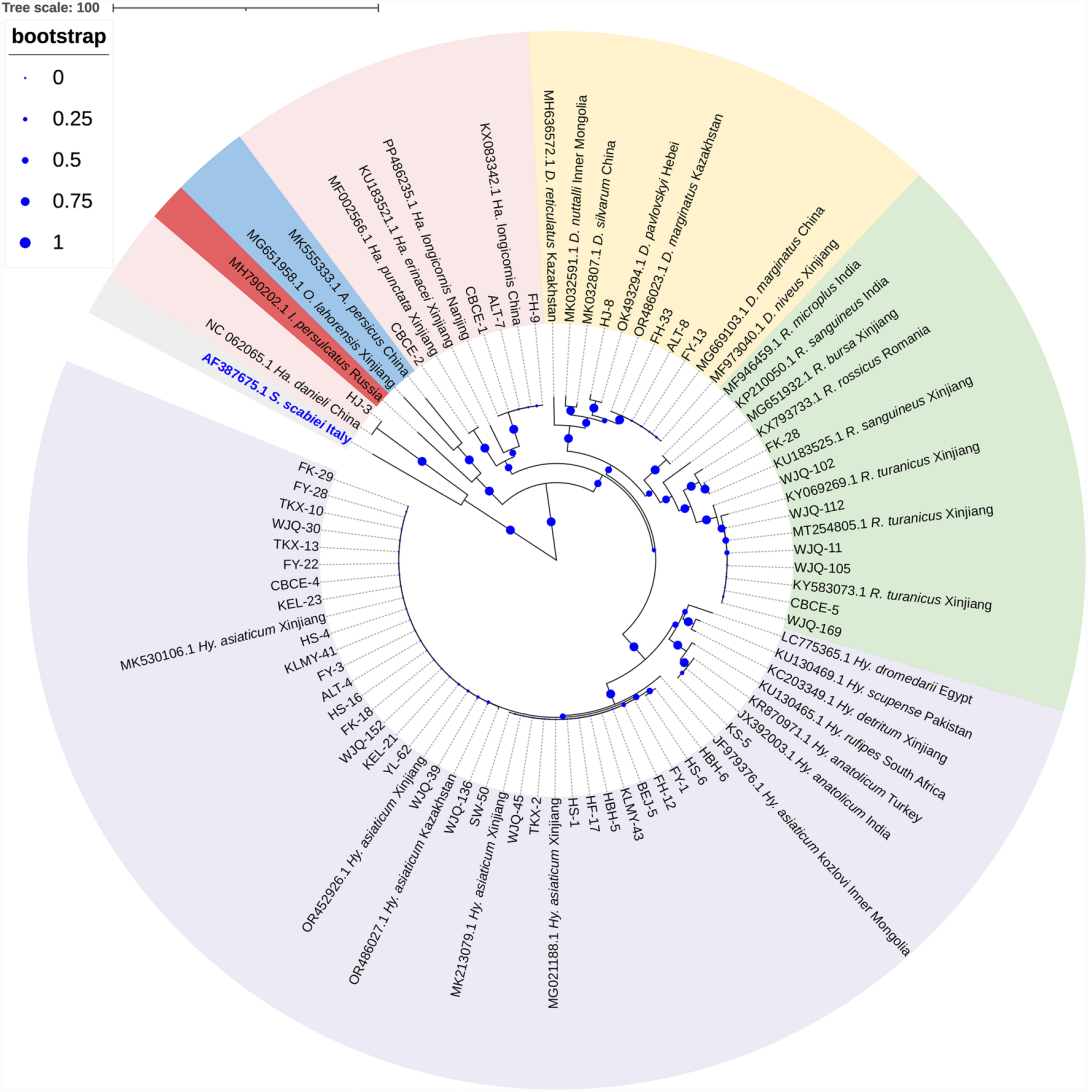
Phylogenetic analysis of tick species based on *16S rRNA* gene sequences. Phylogenetic tree was conducted using the neighbor-joining method under the Tamura 3-parameter model. The robustness of the tree topology was assessed through bootstrap analysis with 1000 pseudoreplicates. In the figure, the purple, green, yellow, pink, red–brown, and blue regions represent species of the *Hyalomma*, *Rhipicephalus Dermacentor*, *Haemaphysalis*, *Ixodes*, and *Argasidae* genera, respectively. Outgroup taxa are labeled in blue text. Bootstrap values are indicated by blue dots on the branches, with dot size proportional to the bootstrap support value (larger dots indicate higher support)

Phylogenetic analysis of *16S rRNA* sequences classified the 48 sequences into five major clades, which corresponded to the *Hyalomma*, *Dermacentor*, *Haemaphysalis* (two subclades), and *Rhipicephalus* genera. Species within the same genus were clustered together, with clear distinction between different genera. Notably, *Ha. danieli* from Hejing County formed a distinct clade, suggesting significant evolutionary divergence within the *Haemaphysalis* genus (Fig. 2 and Supplementary Fig. S1).

### Haplotype diversity analysis of the *16S rRNA* gene in *Hy. asiaticum*

Genetic diversity analysis of 83 mitochondrial *16S rRNA* gene sequences from eight *Hy. asiaticum* populations (including 73 new sequences and 10 sequences retrieved from GenBank, whose detailed information is provided in Additional file 1: Supplementary Table S4) revealed nine distinct haplotypes. Among these haplotypes, five were shared haplotypes (Hap_1 to Hap_2, Hap_6 to Hap_8), while the other four were unique haplotypes (Hap_3 to Hap_5, Hap_9). The overall haplotype diversity (Hd) was 0.734, with a nucleotide diversity (*π*) of 0.00403. Population analysis showed that the Changji population exhibited the lowest haplotype diversity (Hd = 0.333), while the Turpan and Ili populations displayed the highest haplotype diversity (Hd = 1.000). The Changji population showed the lowest nucleotide diversity (*π* = 0.00113), whereas the Tacheng population had the highest nucleotide diversity (*π* = 0.01081; Table 7). These results indicated that the eight populations have high haplotype diversity but relatively low nucleotide diversity, which is a characteristic of tick populations with high genetic variability. Tajima’s D tests revealed negative values for all populations except for the population from Karamay (Table 7), suggesting that the seven populations have undergone expansion, while the population from Karamay may have been restricted by a bottleneck effect.

**Table 7:** Population diversity parameters of* Hy. asiaticum*

Population	No.	Hn	Shared haplotype	Private haplotype	Hd	*π*	Tajima’s D	Fu’s Fs
Bayingolin	22	4	Hap_1, Hap_2, Hap_6, Hap_7	–	0.403	0.00173	−0.98105	−1.428
Turpan	3	3	Hap_1, Hap_2, Hap_8	–	1.000	0.00450	NA	−1.216
Wujiaqu	20	5	Hap_1, Hap_2, Hap_6	Hap_5, Hap_9	0.726	0.00359	−0.16515	−0.958
Changji	6	2	Hap_1, Hap_6	−	0.333	0.00113	−0.93302	−0.003
Karamay	8	2	Hap_2, Hap_6	−	0.536	0.00362	1.44880	2.083
Tacheng	5	2	Hap_2	Hap_4	0.400	0.01081	−1.17432	3.679
Ili	3	3	Hap_1, Hap_2	Hap_3	1.000	0.00450	NA	−1.216
Altay	16	5	Hap_1, Hap_2, Hap_6, Hap_7, Hap_8	–	0.667	0.00363	−0.33859	−1.243
Total	83	9	5	4	0.734	0.00403	−1.50200	−2.467

No*.* number of isolates, *Hn* number of haplotypes, *Hd* haplotype diversity, *π* nucleotide diversity, *NA* test was not applied because the sample size was less than four

**Fig. 3:**
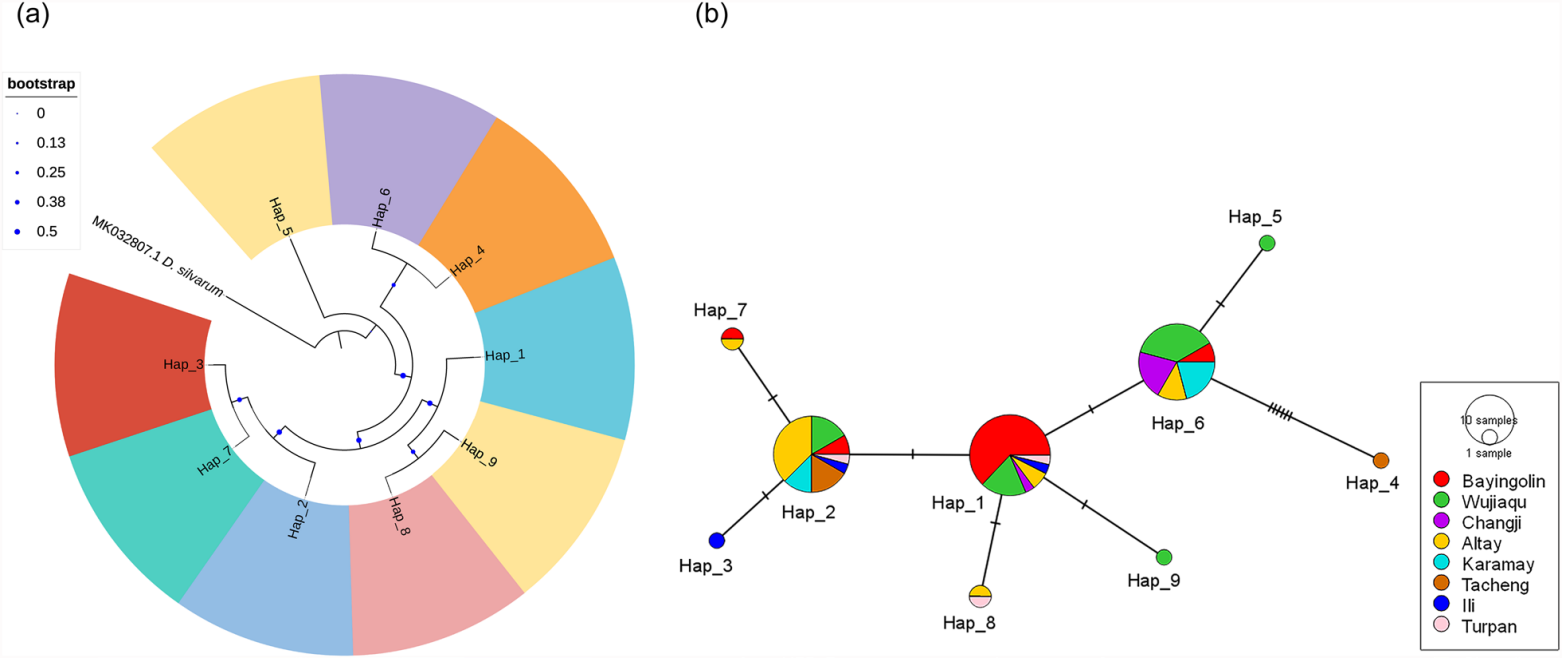
Haplotype phylogenetic tree (**a**) and network (**b**) of *Hy. asiaticum* based on *16S rRNA* gene sequences. **a** Hap_5 and Hap_9, which were derived from the same population, are indicated by the same color, while other haplotypes, representing distinct populations, are displayed in different colors. **b** Haplotype network depicting the distribution of haplotypes across populations, with each color corresponding to a unique population. The size of each circle is proportional to the frequency of the respective haplotype. Transverse lines on the network represent a gene mutation site

Haplotype evolutionary analysis revealed that the eight *Hy. asiaticum* populations in Xinjiang did not form independent lineages, as their phylogenetic relationships exhibited a cross-nested pattern and no significant geographic differentiation (Fig. 3a). The haplotype network analysis also demonstrated that haplotypes from the eight populations did not form distinct lineage structures and were interwoven with each other. Hap_1 occupied the central position in the network, with other haplotypes being distributed peripherally as derived haplotypes, indicating that Hap_1 is a stable haplotype formed during the evolutionary history of *Hy. asiaticum* (Fig. 3b).

### Genetic differentiation of the *16S rRNA* gene in *Hy. asiaticum*

The genetic differentiation index (Fst) among the eight populations of *Hy. asiaticum* in Xinjiang ranged from −0.12113 to 0.63889. The genetic differentiation was the weakest between the Turpan and Altai populations, while it was the strongest between the Changji and Ili populations (Table 8).

**Table 8:** Genetic differentiation coefficients among populations of *Hy. asiaticum*

Population	Bayingolin	Altay	Tacheng	Ili	Wujiaqu	Changji	Karamay	Turpan
Bayingolin	0.00000							
Altay	0.22134	0.00000						
Tacheng	0.38085	0.04920	0.00000					
Ili	0.33425	−0.06040	−0.12113	0.00000				
Wujiaqu	0.15476	0.17778	0.26438	0.25626	0.00000			
Changji	0.52235	0.44512	0.38030	0.63889	0.05927	0.00000		
Karamay	0.30730	0.13041	0.14038	0.22499	−0.04101	0.08682	0.00000	
Turpan	0.07899	−0.01964	0.14038	−0.09091	0.13859	0.55172	0.17799	0.00000

Analysis of molecular variance (AMOVA) revealed that 19.7% of the total genetic variations occurred among populations, while 80.3% of them were variations within populations (Table 9), indicating that the genetic diversity within populations is significantly greater than that between populations.

**Table 9:** Analysis of molecular variance among populations of *Hy. asiaticum*

Source of variation	*df*	Sum of squares	Variance components	Percentage variation (%)
Among populations	7	11.691	0.12172	19.7
Within populations	75	37.176	0.49568	80.3
Total	82	48.867	0.61741	–

### Sequencing and phylogenetic analysis of various tick-borne pathogens

**Fig. 4:**
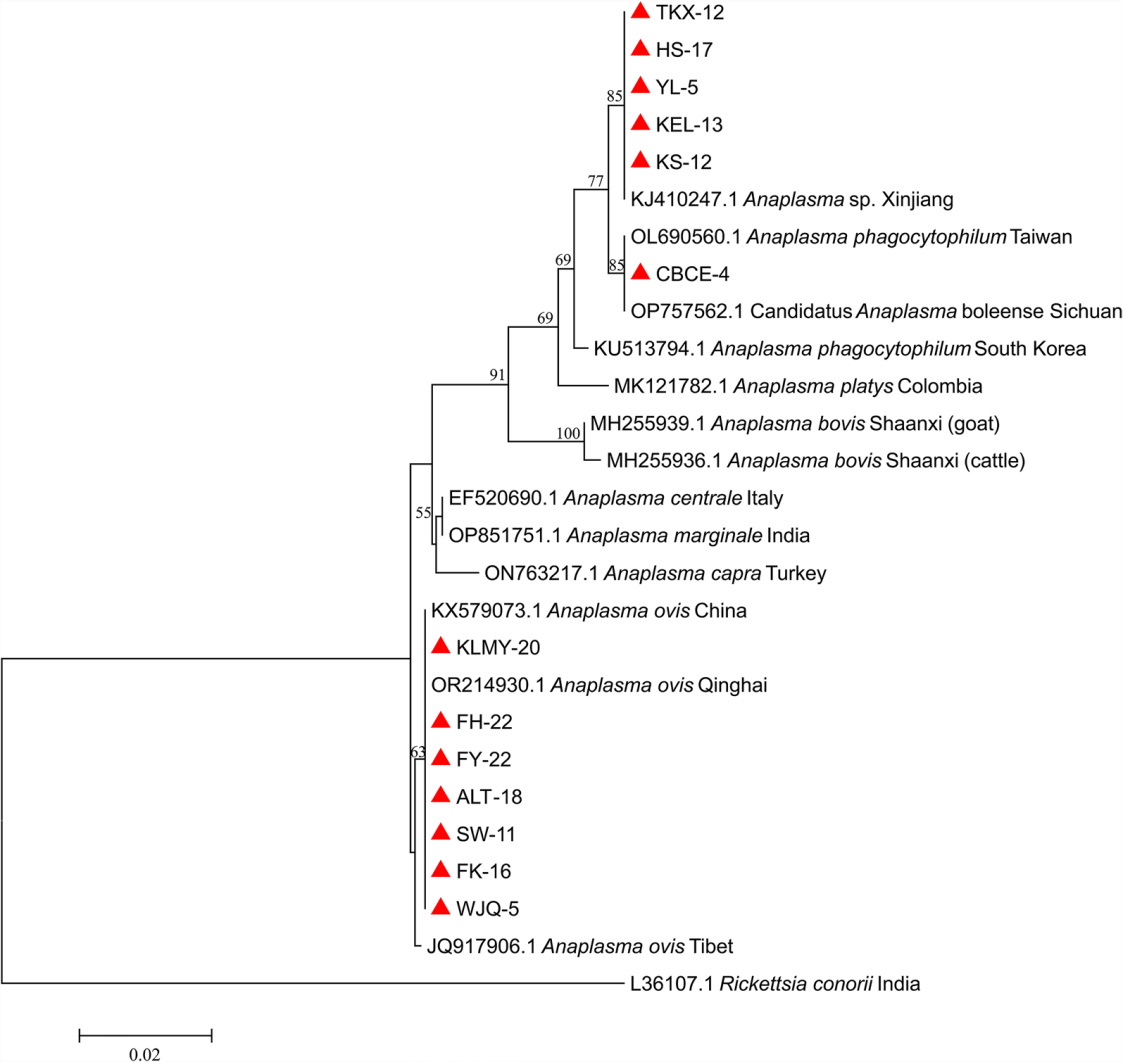
Phylogenetic analysis of piroplasms based on *18S rRNA* gene sequences. The phylogenetic tree was constructed using the neighbor-joining method under the Tamura–Nei parameter model. Sequences obtained from this study are highlighted with red circles in the tree

For the *Anaplasma* genus, three species (*A. ovis*, *Anaplasma* sp., and *A. phagocytophilum*) were identified in the ticks. In the Kashgar, Bayingolin, and Turpan regions, all detected *Anaplasma* species were identified as *Anaplasma* sp., with a 100% identity to *Anaplasma* sp. from Xinjiang (KJ410247.1). The *Anaplasma* species in tick samples from the Wujiaqu, Changji, Karamay, Tacheng, and Altay regions were identified as *A. ovis*. Notably, the *A. ovis* found in Karamay demonstrated 99.8% identity with the Qinghai strain (OR214930.1), while those from other regions exhibited 99.8–100% identity with the Chinese strain (KX579073.1). Furthermore, ticks from the Ili region were found to harbor *Anaplasma*, which was identified as *A. phagocytophilum*, with a 100% identity to the Taiwanese strain (OL690560.1). Phylogenetic analysis of *16S rRNA* gene sequences from *Anaplasma* species with *R. conorii* as the outgroup revealed distinct clustering patterns. The sequences of *A. ovis*, *A. phagocytophilum*, and *Anaplasma* sp. formed distinct monophyletic clades with their respective reference sequences from GenBank. Notably, the geographical variants of *A. ovis* diverged into sister clades, demonstrating potential genetic differentiation among distinct geographical populations (Fig. 4).

**Fig. 5:**
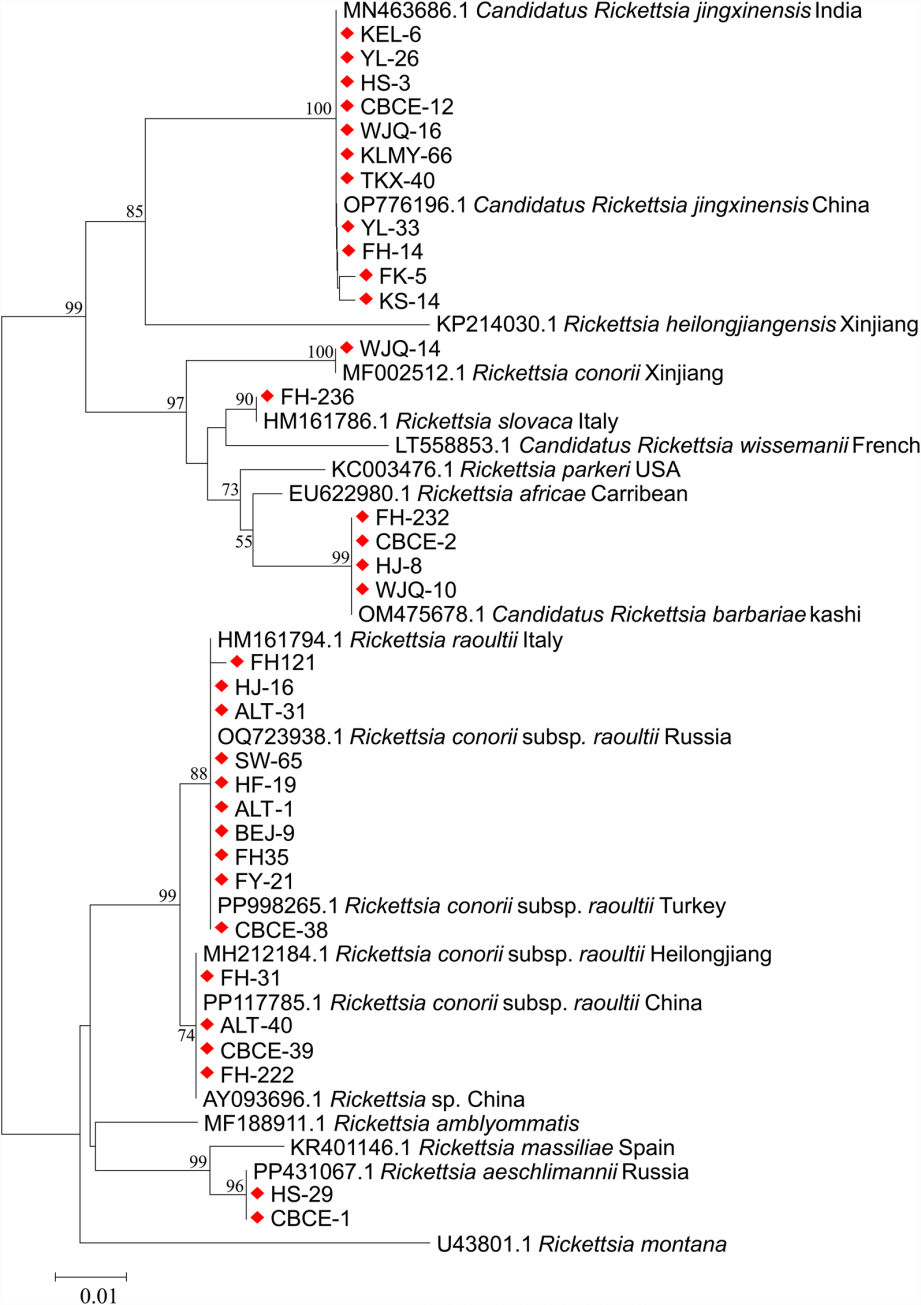
Phylogenetic analysis of *Anaplasma* based on *16S rRNA* gene sequences. The phylogenetic tree was constructed using the neighbor-joining method under the Kimura two-parameter model. Red triangles indicate sequences obtained from this study

For *Rickettsia*, seven species were identified in ticks collected from various regions of Xinjiang, including *R. aeschlimannii*, *R. conorii*, *R. slovaca*, *R. conorii* subsp. *raoultii*, *Rickettsia* sp., *Candidatus R. barbariae*, and *Candidatus R. jingxinensis*. In the Kashgar region, *Candidatus R. jingxinensis* was detected, which exhibited 99.8% nucleotide identity with the Indian strain (MN463686.1). In the Bayingolin region, multiple *Rickettsia* species were identified, including *R. aeschlimannii*, *R. conorii* subsp. *raoultii*, *Candidatus R. barbariae*, and *Candidatus R. jingxinensis*, all of which displayed 100% identity with their respective reference strains, namely the Russian strain of *R. aeschlimannii* (PP431067.1), the Russian strain of *R. conorii* subsp. *raoultii* (OQ723938.1), the Kashi strain of *Candidatus R. barbariae* (OM475678.1), the Indian strain of *Candidatus R. jingxinensis* (MN463686.1), and the Chinese strain of *Candidatus R. jingxinensis* (OP776196.1). In the Turpan region, *Candidatus R. jingxinensis* was detected, which showed a 100% identity with the Indian strain (MN463686.1). In Wujiaqu City, *R. conorii*, *Candidatus R. barbariae*, and *Candidatus R. jingxinensis* were identified, with 100% identity to the Xinjiang strain of *R. conorii* (MF002512.1); the Kashi strain of *Candidatus R. barbariae* (OM475678.1); and the Indian strain of *Candidatus R. jingxinensis* (MN463686.1). In the Changji region and Karamay City, all *Rickettsia* species were identified as *Candidatus R. jingxinensis*, which exhibited 99.8–100% identity with the Indian strain (MN463686.1). In the Tacheng region, *R. conorii* subsp. *raoultii* was detected, showing 100% identity with the Turkish strain (PP998265.1). In the Ili region, a diverse array of *Rickettsia* species was identified, including *R. aeschlimannii*, *R. conorii* subsp. *raoultii*, *Candidatus R. barbariae*, and *Candidatus R. jingxinensis*, all of which demonstrated 100% identity with their reference strains from various regions, including the Russian strain of *R. aeschlimannii* (PP431067.1), the Turkish strain of *R. conorii* subsp. *raoultii* (PP998265.1), the Chinese strain of *R. conorii* subsp. *raoultii* (PP117785.1), the Kashi strain of *Candidatus R. barbariae* (OM475678.1), and the Indian strain of *Candidatus R. jingxinensis* (MN463686.1). In Altay, *R. slovaca*, *Rickettsia* sp., *Candidatus R. barbariae*, *Candidatus R. jingxinensis*, and *R. conorii* subsp. *raoultii* were detected, where the former four species had 100% identity to the Italian strain of *R. slovaca* (HM161786.1), the Chinese strain of *Rickettsia* sp. (AY093696.1), the Kashi strain of *Candidatus R. barbariae* (OM475678.1), and the Chinese strain of *Candidatus R. jingxinensis* (OP776196.1), respectively, while *R. conorii* subsp. *raoultii* showed 100% identity to the Turkish strain (PP998265.1), the Heilongjiang strain (MH212184.1), the Chinese strain (PP117785.1), and the Russian strain (OQ723938.1). The phylogenetic tree constructed on the basis of the *opmA* gene of *Rickettsia* revealed that the seven *Rickettsia* species obtained in this study were clustered in the same clades with their corresponding reference strains, and the bootstrap support values ranged from 74.0% to 100%. Notably, the *R. conorii* subsp. *raoultii* identified in this study formed a clade with *R. raoultii*, while exhibiting a distant phylogenetic relationship with *R. conorii*. The Chinese strain of *R. conorii* subsp. *raoultii* formed a sister clade with strains from other geographical origins, suggesting significant genetic differentiation among populations of this subspecies from different regions (Fig. 5).

**Fig. 6:**
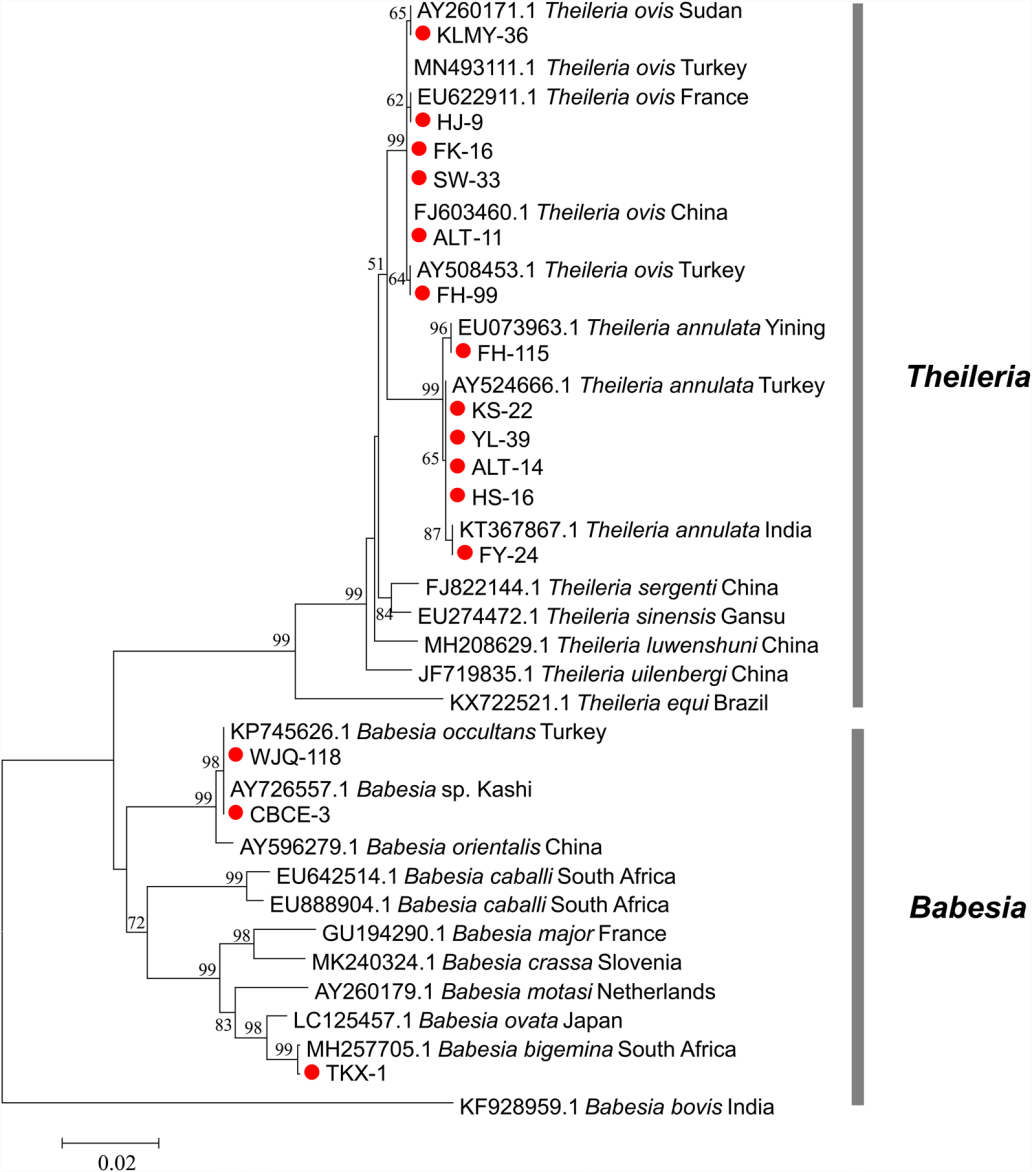
Phylogenetic analysis of *Rickettsia* based on *ompA* gene sequences. The phylogenetic tree was performed using the neighbor-joining method under the Tamura three-parameter model. Red diamonds indicate sequences obtained from this study

For piroplasms, based on the *18S rRNA* gene, the 98 piroplasm-positive tick samples were infected with five piroplasm species (*T. annulata*, *T. ovis*, *B. bigemina*, *B. occultans*, and *Babesia* sp.). In the Kashgar region, the detected piroplasms were identified as *T. annulata*, which showed a 100% identity with the Turkish strain of *T. annulata* (AY524666.1). In the Bayingolin region, the detected piroplasms included *T. annulata* and *T. ovis*, both of which showed a 100% identity to the Turkish strain of *T. annulata* (AY524666.1) and the French strain of *T. ovis* (EU622911.1), respectively. In the Turpan region, *B. bigemina* was detected in the ticks, which shared 99.9% identity with the South African strain of *B. bigemina* (MH257705.1). In Wujiaqu City, ticks contained *B. occultans*, which showed 100% identity with the Turkish strain of *B. occultans* (KP745626.1). In the Changji region, Karamay City, and Tacheng region, the detected piroplasms were all identified as *T. ovis*, displaying 100% identity with the Turkish strain (MN493111.1), the Sudanese strain (AY260171.1), and the Turkish strain (MN493111.1), respectively. In the Ili region, ticks were found to harbor *Babesia* sp., exhibiting 100% identity with Kashi strain (AY726557.1). Finally, ticks in the Altay region showed the presence of both *T. annulata* and *T. ovis*, with *T. annulata* showing 100% identity to the Turkish strain (AY524666.1), the Yining strain (EU073963.1), and the Indian strain (KT367867.1); and *T. ovis* showing 100% identity to the Chinese strain (FJ603460.1) and the Turkish strain (AY508453.1). Phylogenetic analysis based on *18S rRNA* gene sequences of piroplasm species revealed that the five piroplasm species identified in this study were clustered into monophyletic clades together with their corresponding reference strains. The tree topology could be divided into three major clades: clade I, which exclusively comprises a *Theileria* species, including *T. annulata* and *T. ovis* obtained in this study, along with reference sequences of *T. annulata*, *T. ovis*, and other *Theileria* spp. from the GenBank; clade II, which encompasses *Babesia* species, including *B. bigemina*, *Babesia* sp., and *B. occultans* identified in this study, as well as reference strains of *B. bigemina*, *Babesia* sp., *B. occultans*, and other *Babesia* spp. from GenBank; and clade III, which is represented solely by the *B. bovis* Indian reference strain. This pronounced segregation underscored substantial genetic divergence between the *Theileria* and *Babesia* genera (Fig. 6).

## Discussion

Xinjiang is characterized by a high diversity of ticks, with the recording of 48 species across two families and eight genera since the initiation of tick fauna surveys in the 1950 s [33]. About three decades ago, *I. persulcatus*, *D. nuttalli*, *Hy. asiaticum*, *D. marginatus*, and *D. niveus* were dominant in the tick communities [34]. However, there have been dynamic shifts in prevalent tick species in recent decades. Initial surveys across 14 northern counties revealed that *Hy. asiaticum*, *Ha. punctata*, *D. nuttalli*, *D. marginatus*, and *R. turanicus* are predominant ticks infesting livestock [35]. Subsequent expansion of the scope to 35 counties (particularly border areas) revealed a distinct dominance of *R. turanicus*, *D. niveus*, *Hy. asiaticum*, and *D. marginatus* in domestic animals [17]. More recently, a comprehensive tick distribution dataset, which involved 108 counties and was synthesized from literature databases and historical records, identified *Hy. asiaticum*, *R. turanicus*, *D. marginatus*, and *Ha. punctata* as currently predominant tick species [33]. In this study, the 1093 ticks collected from 19 sampling sites across nine regions of Xinjiang were classified into nine species spanning four genera. *Hy. asiaticum* (72.0%, 787/1,093) was identified as the dominant species, followed by *D. marginatus* (15.6%, 171/1,093) and *R. turanicus* (5.4%, 59/1,093), which is slightly different from previous reports. *Hy. asiaticum* was collected in all regions except for Kashgar in this study, indicating that there is a wide distribution of this tick species in the Xinjiang region. This finding is consistent with earlier reports on the presence of *Hy. asiaticum* in the Junggar Basin, eastern Xinjiang basins (Yanqi, Turpan, Hami), the Tarim Basin, and parts of northern Xinjiang [36], suggesting minimal changes in its geographic distribution. Conversely, *D. marginatus* was only collected in the Altai region in this study, which contrasts with previous reports of its widespread distribution in the Tianshan Mountains, the Ili River Valley, the Altai Mountains, the Junggar Basin, and the Tarim Basin [37]. *R. turanicus* was detected in Wujiaqu and Ili, and its primary distribution was historically concentrated in the Tarim Basin and parts of the Tianshan Mountains and Ili River Valley [17]. However, recent findings in Fukang, Jimsar, Changji, Hutubi [38], and the Sixth Division of the Xinjiang Production and Construction Corps [39] have suggested an expanding trend of the geographic range for this species. Notably, *Ha. longicornis*—a species widely distributed across Asia and the Pacific, including China, Russia, South Korea, Japan, Australia, New Zealand, and the South Pacific Islands [40], was detected in Ili and Altai. This species was previously reported in China’s northern and central provinces [41] and had only one prior record in Xinjiang (Changji) [42]. Its expanded detection signals a potential trend of northward spread of this species in Xinjiang. These findings highlight the shifting patterns of dominant tick species and their geographic distribution across Xinjiang. Given the role of ticks as vectors for pathogens, such spatial dynamics may drive the spread of tick-borne diseases. Hence, continuous surveillance of tick distribution and population trends is critical for mitigating public health risks associated with these arthropod-borne pathogens.

*Hyalomma asiaticum* is the dominant tick species in Xinjiang widely reported across the region [33–36]. However, there are still critical knowledge gaps regarding its haplotype diversity, genetic differentiation, and population expansion dynamics. Here, we analyzed the mitochondrial *16S rRNA* gene sequences from eight *Hy. asiaticum* populations in Xinjiang to assess their genetic diversity and population structure. The results revealed high haplotype diversity (Hd = 0.734) and low nucleotide diversity (*π* = 0.00403) across the eight populations. According to the genetic diversity classification by Grant et al. [43], these populations are characterized by high haplotype diversity and low nucleotide diversity, indicating a stable genetic structure. This pattern is in line with the findings of Zhu et al. [44] on the genetic diversity of *D. niveus* in Xinjiang. The observed high haplotype diversity suggests that these populations have undergone long-term development and evolution, resulting in the formation of a large and stable population. Genetic differentiation index (Fst) and genetic distance are essential metrics for evaluating the population divergence. Theoretically, the Fst values range from −1 to 1, with a value closer to zero indicating lower differentiation between populations [45]. In this study, the Fst values among the eight *Hy. asiaticum* populations ranged from −0.12113 to 0.63889, indicating that most populations have moderate-to-high genetic differentiation. Notably, the populations in Turpan and Ili showed moderate-to-high differentiation from those in other regions, which may be attributable to limited sample sizes (Table 7). Conversely, differentiation between other populations may stem from geographic isolation caused by the mountainous terrain of Xinjiang, which restricts the flow of genes.

Climate change tends to drive the geographic expansion of tick populations and increases global transmission risk of tick-borne diseases. However, there has been rather limited research on tick-borne pathogens in northwest China [35]. In this study, we conducted surveillance on tick-borne bacteria and piroplasms in ticks collected from 19 sampling sites in Xinjiang, northwest China. The results revealed an overall prevalence of 9.3% (102/1093) for *Anaplasma*, 18.1% (198/1093) for *Rickettsia*, and 9.0% (98/1093) for piroplasms. The high prevalence rate of *Rickettsia* aligns with prior reports (10.59%) for the southern edge of the Gurbantünggüt Desert in northern Xinjiang [46]. Three *Anaplasma* species, seven *Rickettsia* species, and five piroplasm species were identified. Among the identified *Anaplasma* species, *A. ovis* was the most prevalent, which was detected in ticks from Altay, Tacheng, Karamay, Changji, and Wujiaqu. *Anaplasma ovis*, which was first identified in sheep in 1912 [47], has been recognized as a potential zoonotic pathogen [48]. In China, *A. ovis* has a wide distribution and infects various animal hosts [49, 50]. While earlier studies have failed to detect *A. ovis* in *D. nuttalli* and *D. marginatus* ticks from 11 Xinjiang counties [51], subsequent molecular studies confirmed its presence in *Hy. anatolicum*, *D. nuttalli*, and *D. marginatus* [19]. This study further expanded the range of its vectors to include *Hy. asiaticum*, *R. turanicus*, and *D. marginatus*. *R. raoultii* was predominant among the detected *Rickettsia* species. *R. raoultii* is a novel species within the *Rickettsia* genus and was first identified in *Dermacentor* ticks in Russia and France in 2008 [52]. Human infections caused by *R. raoultii* have been reported globally [53]. In China, *R. raoultii* was first detected in Xinjiang in 2012 [54], with subsequent reports in *D. marginatus* from Habahe County (17.6%) in 2016 [55] and in *D. marginatus* and *D. silvarum* from Zhaosu and Hejing Counties (36.8% prevalence in Hejing) in 2021 [56]. Our detection of *R. raoultii* in multiple tick species across Altay, Tacheng, Ili, and Bayingolin, highlights the need for further studies to assess its risk level and prevent human infections. For piroplasms, *T. ovis* was the dominant species, which is currently restricted to Xinjiang, and its primary vector is *Hy. anatolicum* [57]. A survey in 2017 found that *T. ovis* is the predominant *Theileria* species infecting sheep in southern Xinjiang [58], and its nucleic acid was detected in *R. sanguineus*, *Ha. punctata*, and *Hy. rufipes* in 2022 [59], suggesting its potential emergence as a major *Theileria* species in this region. Notably, we detected the nucleic acid of *B. occultans* in ticks, a species rarely reported in China. Since its first detection in *Hy. asiaticum* ticks from Alashankou on the China–Kazakhstan border in 2016 [60], *B. occultans* has been detected in *D. nuttalli* from Jimunai County; *Hy. anatolicum* from Artux City; *Hy. asiaticum* from Fuyun and Burqin Counties; and *D. marginatus* from Tacheng City [59, 61]. Our discovery of *B. occultans* in *R. turanicus* from Wujiaqu indicates the expansion of both its vector range and geographic distribution, suggesting necessity of strengthening surveillance on *B. occultans.*

However, this study still has several limitations that should be noted. First, the sample sizes for certain tick species and specific regions were small, which may restrict the comprehensive detection of tick-borne pathogens and the comparative analysis of pathogen prevalence across different areas. Second, we did not collect animal blood samples to correlate the prevalence of tick-borne pathogens detected with potential host infections. For instance, while *B. occultans* was detected in ticks collected from sheep, the positive PCR results could not distinguish whether the pathogen originated from degraded sheep blood in the tick gut or from an established *B. occultans* infection within the tick. Therefore, the role of ticks in the transmission of *B. occultans* requires further investigation.

## Conclusions

This study elucidates the diversity and distribution of tick species across nine regions in Xinjiang, China and identifies the presence and infection status of multiple tick-borne pathogens. Furthermore, it provides preliminary evidence for the genetic differentiation and population expansion of distinct *Hy. asiaticum* populations in this region. The findings contribute to a deeper understanding of the epidemiology of ticks and tick-borne pathogens, offering a foundation for future research on them and targeted control strategies.

## Supplementary Information

Additional file 1: Table S1. Primers used for tick species identification and screening of tick-borne pathogens. Table S2. Sequencing data obtained in this study. Table S3. Reference sequence information used for phylogenetic tree construction in the study. Table S4. Reference sequence information for haplotype phylogenetic tree construction of *Hy. asiaticum.* Fig. S1. Phylogenetic analysis of tick species based on *16S rRNA* gene sequences.

## Data Availability

Data are provided within the manuscript or supplementary information files.

## Abbreviations

PCR: Polymerase chain reaction
SFG: Spotted fever group
TG: Typhus group
AG: Ancestral group
TRG: Transitional group
HGA: Human granulocytic anaplasmosis
HME: Human monocytic ehrlichiosis
TBE: Tick-borne encephalitis
CCHF: Crimean–Congo hemorrhagic fever
PBS: Phosphate-buffered saline
NJ: Neighbor-joining
Hn: Number of haplotypes
Hd: Haplotype diversity
*π*: Nucleotide diversity
Fst: F statistics
AMOVA: Analysis of molecular variance
*χ*^2^: Chi-squared
TBPs: Tick-borne pathogens
