# Supplementary material for: Molecular characterization of tick-borne bacterial and protozoan pathogens in parasitic ticks from Xinjiang, China
Source: Parasit Vectors. 2025 Jun 4;18:207. doi: 10.1186/s13071-025-06857-1 (PMC12139197; doi:10.1186/s13071-025-06857-1)

***Additional file***

**Molecular characterization of tick-borne bacterial and protozoan pathogens**

**in parasitic ticks from Xinjiang, China**

**Bingjie Wang^1, 2^, Zhiqiang Liu^3^, Shiying Zhu^1, 2^, Jinchao Zhang^1, 2^, Wenwen Qi^4^, Jianyu Wang^1, 2^, Dongfang Li^1, 2^, Lan He^1, 2, 5*^,**

**Junlong Zhao^1, 2, 5*^**

***Correspondence:** zhaojunlong@mail.hzau.edu.cn

[helan@mail.hzau.edu.cn](mailto:helan@mail.hzau.edu.cn)

**Table S1** Primers used for tick species identification and screening of tick-borne pathogens

| Species | Target gene | Primer name | Primer sequence (5′→3′) | Annealing temperature (℃) | Product size (bp) | References |
| --- | --- | --- | --- | --- | --- | --- |
| Tick | 16S rRNA | 16S-F | CTGCTCAATGATTTTTTAAATTGCTGTGG | 54 | 460 | [34] |
|  |  | 16S-R | CCGGTCTGAACTCAGATCAAGT |  |  |  |
| *Amaplasma/Ehrlichia* | 16S rRNA | Eh-out1 F | TTGAGAGTTTGATCCTGGCTCAGAACG | 50 | 1140 | [35] |
|  |  | Eh-3-17 R | TAAGGTGGTAATCCAGC |  |  |  |
|  |  | Eh-out1 F | TTGAGAGTTTGATCCTGGCTCAGAACG | 55 | 650 |  |
|  |  | Eh-out2 R | CACCTCTACACTAGGAATTCCGCTATC |  |  |  |
| *Rickettsia* | *ompA* | Rr190.70 F | ATGGCGAATATTTCTCCAAAA | 50 | 632 | [36] |
|  |  | Rr190.701 R | GTTCCGTTAATGGCAGCATCT |  |  |  |
|  |  | Rr190.70 F | ATGGCGAATATTTCTCCAAAA | 55 | 533 |  |
|  |  | Rr190.602n R | AGTGCAGCATTCGCTCCCCCT |  |  |  |
| *Borrelia burgdorferi* | 5S~23S rRNA | 23SN1 | CGACCTTCTTCGCCTTAAAGC | 52 | 412 | [37] |
|  |  | 23SC1 | TAAGCTGACTAATACTAATTACCC |  |  |  |
|  |  | 23SN2 | CTGCGAGTTCGCGGGAGA | 59 | 226-266 |  |
|  |  | 5SCB | TCCTAGGCATTCACCATA |  |  |  |
| Piroplasma | 18S rRNA | Nbab1F | AAGCCATGCATGTCTAAGTATAAGCTTTT | 59 | 1600 | [38] |
|  |  | Nbab1R | CTTCTCCTTCCTTTAAGTGATAAGGTTCAC |  |  |  |

**Table S2** Sequencing data obtained in this study

| Species | Numbering in the text | Geographic region | Accession number |
| --- | --- | --- | --- |
| *Hy. anatolicum* | KS-5 | Kashgar | PV219193 |
| *Hy. asiaticum* | Hap_1 | Korla、Yuli、Hoxud、Toksun、Wujiaqu、Fukang、Karamay、Shawan、Hefeng、Qapqal、Altay、Fuyun、Fuhai、Burqin、Habahe | PV219194 |
| *Hy. asiaticum* | Hap_2 |  | PV219195 |
| *Hy. asiaticum* | Hap_3 |  | PV219196 |
| *Hy. asiaticum* | Hap_4 |  | PV219197 |
| *Hy. asiaticum* | Hap_5 |  | PV219198 |
| *Hy. asiaticum* | Hap_6 |  | PV219199 |
| *Hy. asiaticum* | Hap_7 |  | PV219200 |
| *Hy. asiaticum* | Hap_8 |  | PV219201 |
| *Hy. asiaticum* | Hap_9 |  | PV219202 |
| *R. turanicus* | WJQ-11 | Wujiaqu | PV219203 |
| *R. turanicus* | WJQ-102 | Wujiaqu | PV219204 |
| *R. turanicus* | WJQ-105 | Wujiaqu | PV219205 |
| *R. turanicus* | WJQ-112 | Wujiaqu | PV219206 |
| *R. turanicus* | WJQ-169 | Wujiaqu | PV219207 |
| *R. turanicus* | CBCE-5 | Qapqal | PV219208 |
| *R. sanguineus* | FK-28 | Fukang | PV219209 |
| *Ha. longicornis* | CBCE-1 | Qapqal | PV219210 |
| *Ha. danieli* | HJ-3 | Hejing | PV219211 |
| *Ha. punctata* | CBCE-2 | Qapqal | PV219212 |
| 1. *pavlovskyi* | HJ-8 | Hejing | PV219213 |
| *D. marginatus* | FH-33 | Fuhai | PV219214 |
| *Anaplasma sp.* | KS-12 | Kashgar | PV202447 |
| *Anaplasma sp.* | KEL-13 | Korla | PV202448 |
| *Anaplasma sp.* | YL-5 | Yuli | PV202449 |
| *Anaplasma sp.* | TKX-12 | Toksun | PV202450 |
| *Anaplasma sp.* | HS-17 | Hoxud | PV202451 |
| *A. ovis* | WJQ-5 | Wujiaqu | PV202452 |
| *A. ovis* | FK-16 | Fukang | PV202453 |
| *A. ovis* | SW-11 | Shawan | PV202454 |
| *A. ovis* | ALT-18 | Altay | PV202455 |
| *A. ovis* | FY-22 | Fuyun | PV202456 |
| *A. ovis* | FH-22 | Fuhai | PV202457 |
| *A. ovis* | KLMY-20 | Karamay | PV202458 |
| *A. phagocytophilum* | CBCE-4 | Qapqal | PV202459 |
| *T. annulata* | KS-22 | Kashgar | PV202460 |
| *T. annulata* | YL-39 | Yuli | PV202461 |
| *T. annulata* | ALT-14 | Altay | PV202462 |
| *T. annulata* | HS-16 | Hoxud | PV202463 |
| *T. annulata* | FH-115 | Fuhai | PV202464 |
| *T. annulata* | FY-24 | Fuyun | PV202465 |
| *B. bigemina* | TKX-1 | Toksun | PV202466 |
| *B. occultans* | WJQ-118 | Wujiaqu | PV202467 |
| *T. ovis* | FK-16 | Fukang | PV202468 |
| *T. ovis* | SW-33 | Shawan | PV202469 |
| *T. ovis* | ALT-11 | Altay | PV202470 |
| *T. ovis* | FH-99 | Fuhai | PV202471 |
| *T. ovis* | HJ-9 | Hejing | PV202472 |
| *T. ovis* | KLMY-36 | Karamay | PV202473 |
| *Babesia sp.* | CBCE-3 | Qapqal | PV202474 |

**Table S3** Reference sequence information used for phylogenetic tree construction in the study

| Genus | Species | Geographic region | Accession number |
| --- | --- | --- | --- |
| *Hyalomma* | *Hy. anatolicum* | Turkey | KR870971.1 |
|  | *Hy. anatolicum* | India | JX392003.1 |
|  | *Hy. asiaticum* | Kazakhstan | OR486027.1 |
|  | *Hy. asiaticum* | Xinjiang | OR452926.1 |
|  | *Hy. asiaticum* | Xinjiang | MK530106.1 |
|  | *Hy. asiaticum* | Xinjiang | MG021188.1 |
|  | *Hy. asiaticum* | Xinjiang | MK213079.1 |
|  | *Hy. asiaticum* | Inner Mongolia | JF979376.1 |
|  | *Hy. detritum* | Xinjiang | KC203349.1 |
|  | *Hy. dromedarii* | Egypt | LC775365.1 |
|  | *Hy. rufipes* | South Africa | KU130465.1 |
|  | *Hy. scupense* | Pakistan | KU130469.1 |
| *Dermacentor* | *D. marginatus* | China | MG669103.1 |
|  | *D. marginatus* | Kazakhstan | OR486023.1 |
|  | *D. niveus* | Xinjiang | MF973040.1 |
|  | *D. nuttalli* | Inner Mongolia | MK032591.1 |
|  | *D. pavlovskyi* | Hebei | OK493294.1 |
|  | *D. reticulatus* | Kazakhstan | MH636572.1 |
|  | *D. silvarum* | China | MK032807.1 |
| *Haemaphysalis* | *Ha. danieli* | China | NC_062065.1 |
|  | *Ha. erinacei* | Xinjiang | KU183521.1 |
|  | *Ha. longicornis* | China | KX083342.1 |
|  | *Ha. longicornis* | Nanjing | PP486235.1 |
|  | *Ha. punctata* | Xinjiang | MF002566.1 |
| *Rhipicephalus* | *R. bursa* | Xinjiang | MG651932.1 |
|  | *R. microplus* | India | MF946459.1 |
|  | *R. rossicus* | Romania | KX793733.1 |
|  | *R. sanguineus* | Xinjiang | KU183525.1 |
|  | *R. sanguineus* | India | KP210050.1 |
|  | *R. turanicus* | Xinjiang | MT254805.1 |
|  | *R. turanicus* | Xinjiang | KY583073.1 |
|  | *R. turanicus* | Xinjiang | KY069269.1 |
| *Ixodes* | *Ixodes persulcatus* | Russia | MH790202.1 |
| *Argas* | *Argas persicus* | China | MK555333.1 |
| *Ornithodoros* | *Ornithodoros lahorensis* | Xinjiang | MG651958.1 |
| *Sarcoptes* | *Sarcoptes scabiei* | Italy | AF387675.1 |
| *Theileria* | *T. annulata* | Turkey | AY524666.1 |
|  | *T. annulata* | India | KT367867.1 |
|  | *T. annulata* | Yining | EU073963.1 |
|  | *T. equi* | Brazil | KX722521.1 |
|  | *T. luwenshuni* | China | MH208629.1 |
|  | *T. ovis* | Turkey | MN493111.1 |
|  | *T. ovis* | Turkey | AY508453.1 |
|  | *T. ovis* | France | EU622911.1 |
|  | *T. ovis* | Sudan | AY260171.1 |
|  | *T. ovis* | China | FJ603460.1 |
|  | *T. sergenti* | China | FJ822144.1 |
|  | *T. sinensis* | Gansu | EU274472.1 |
|  | *T. uilenbergi* | China | JF719835.1 |
| *Babesia* | *B. bigemina* | South Africa | MH257705.1 |
|  | *B. bovis* | India | KF928959.1 |
|  | *B. caballi* | South Africa | EU642514.1 |
|  | *B. caballi* | South Africa | EU888904.1 |
|  | *B. crassa* | Slovenia | MK240324.1 |
|  | *B. major* | France | GU194290.1 |
|  | *B. motasi* | Netherlands | AY260179.1 |
|  | *B. occultans* | Turkey | KP745626.1 |
|  | *B. orientalis* | China | AY596279.1 |
|  | *B. ovata* | Japan | LC125457.1 |
|  | *Babesia* sp. | Kashi | AY726557.1 |
| *Anaplasma* | *A. bovis* | Shaanxi | MH255939.1 |
|  | *A. bovis* | Shaanxi | MH255936.1 |
|  | *A. capra* | Turkey | ON763217.1 |
|  | *A. centrale* | Italy | EF520690.1 |
|  | *A. marginale* | India | OP851751.1 |
|  | *A. ovis* | China | KX579073.1 |
|  | *A. ovis* | Qinghai | OR214930.1 |
|  | *A. ovis* | Tibet | JQ917906.1 |
|  | *A. phagocytophilum* | Taiwan | OL690560.1 |
|  | *A. phagocytophilum* | South Korea | KU513794.1 |
|  | *A. platys* | Colombia | MK121782.1 |
|  | *Anaplasma* sp. | Xinjiang | KJ410247.1 |
|  | *Candidatus A. boleense* | Sichuan | OP757562.1 |
| *Rickettsia* | *R. aeschlimannii* | Russia | PP431067.1 |
|  | *R. africae* | Carribean | EU622980.1 |
|  | *R. amblyommatis* | Brazil | MF188911.1 |
|  | *R. conorii* | India | L36107.1 |
|  | *R. conorii* | Xinjiang | MF002512.1 |
|  | *R. conorii* subsp*. raoultii* | Turkey | PP998265.1 |
|  | *R. conorii* subsp. *raoultii* | Russia | OQ723938.1 |
|  | *R. conorii* subsp. *raoultii* | China | PP117785.1 |
|  | *R. conorii* subsp. *raoultii* | Heilongjiang | MH212184.1 |
|  | *R. heilongjiangensis* | Xinjiang | KP214030.1 |
|  | *R. massiliae* | Spain | KR401146.1 |
|  | *R. montana* | N | U43801.1 |
|  | *R. parkeri* | USA | KC003476.1 |
|  | *R. raoultii* | Italy | HM161794.1 |
|  | *R. slovaca* | Italy | HM161786.1 |
|  | *Rickettsia* sp. | China | AY093696.1 |
|  | *Candidatus R. barbariae* | kashi | OM475678.1 |
|  | *Candidatus R. jingxinensis* | India | MN463686.1 |
|  | *Candidatus R. jingxinensis* | China | OP776196.1 |
|  | *Candidatus R. wissemanii* | French | LT558853.1 |

N: No information provided in the NCBI or references

**Table S4** Reference sequence information for haplotype phylogenetic tree construction of *Hy. asiaticum*

| Genus | Species | Geographic region | Accession number |
| --- | --- | --- | --- |
| *Hyalomma* | *Hy. asiaticum* | Yuli | KF527442.1 |
|  | *Hy. asiaticum* | Yuli | KF527439.1 |
|  | *Hy. asiaticum* | Fuhai | MK139679.1 |
|  | *Hy. asiaticum* | Fuhai | MK213078.1 |
|  | *Hy. asiaticum* | Buerjin | MF973043.1 |
|  | *Hy. asiaticum* | Buerjin | MG021188.1 |
|  | *Hy. asiaticum* | Shawan | MG669106.1 |
|  | *Hy. asiaticum* | Kuitun | MK213080.1 |
|  | *Hy. asiaticum* | Kuitun | MK213079.1 |
|  | *Hy. asiaticum* | Tacheng | MG021191.1 |

**Fig. S1** Phylogenetic analysis of tick species based on 16S rRNA gene sequences


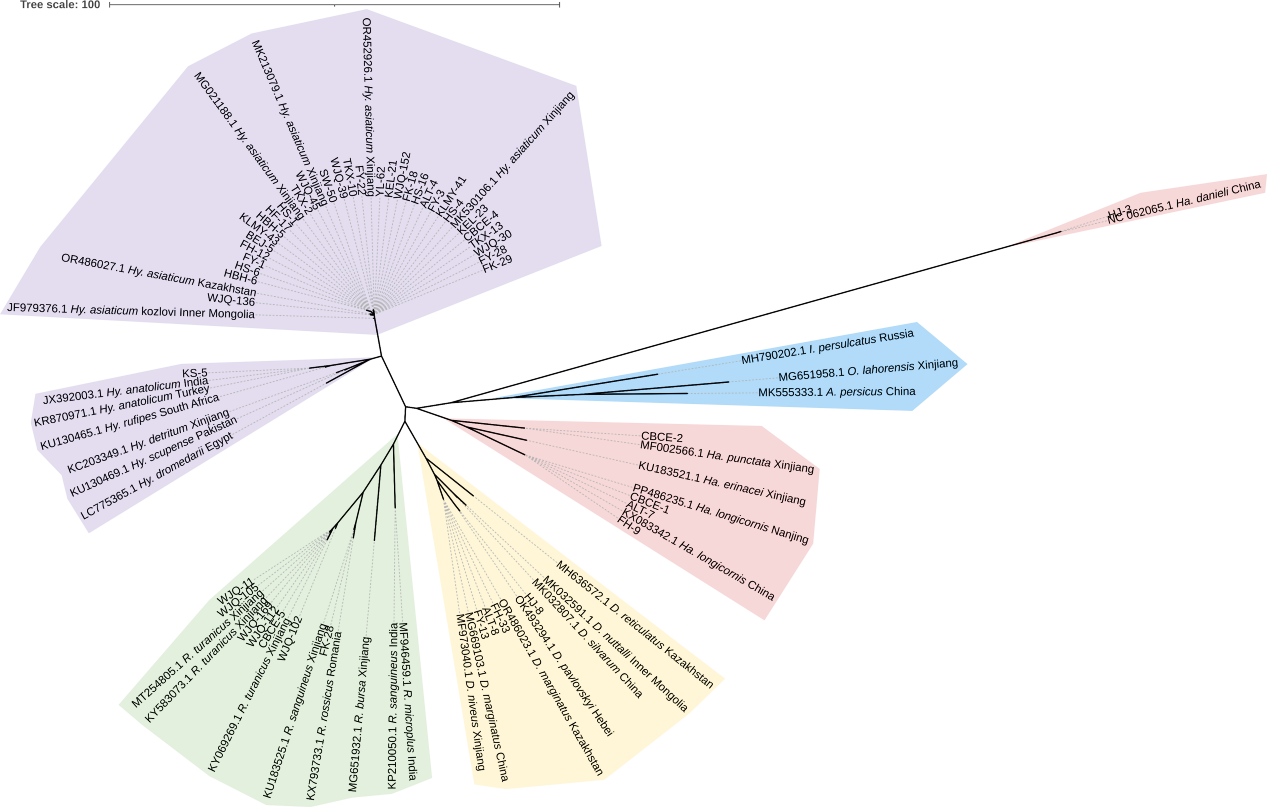

Supplement: Supplementary file 1 — Additional file 1: Table S1. Primers used for tick species identification and screening of tick-borne pathogens. Table S2. Sequencing data obtained in this study. Table S3. Reference sequence information used for phylogenetic tree construction in the study. Table S4. Reference sequence information for haplotype phylogenetic tree construction of Hy. asiaticum. Fig. S1. Phylogenetic analysis of tick species based on 16S rRNA gene sequences. [file 13071_2025_6857_MOESM1_ESM.docx]
